# Presence of thiamine pyrophosphate in mammalian peroxisomes

**DOI:** 10.1186/1471-2091-8-10

**Published:** 2007-06-27

**Authors:** Patrizia Fraccascia, Mieke Sniekers, Minne Casteels, Paul P Van Veldhoven

**Affiliations:** 1 Department of Molecular Cell Biology, Division of Pharmacology, LIPIT, Katholieke Universiteit Leuven, O&N1, Herestraat 49, box 601, 3000 Leuven, Belgium

## Abstract

**Background:**

Thiamine pyrophosphate (TPP) is a cofactor for 2-hydroxyacyl-CoA lyase 1 (HACL1), a peroxisomal enzyme essential for the α-oxidation of phytanic acid and 2-hydroxy straight chain fatty acids. So far, HACL1 is the only known peroxisomal TPP-dependent enzyme in mammals. Little is known about the transport of metabolites and cofactors across the peroxisomal membrane and no peroxisomal thiamine or TPP carrier has been identified in mammals yet. This study was undertaken to get a better insight into these issues and to shed light on the role of TPP in peroxisomal metabolism.

**Results:**

Because of the crucial role of the cofactor TPP, we reanalyzed its subcellular localization in rat liver. In addition to the known mitochondrial and cytosolic pools, we demonstrated, for the first time, that peroxisomes contain TPP (177 ± 2 pmol/mg protein). Subsequently, we verified whether TPP could be synthesized from its precursor thiamine, *in situ*, by a peroxisomal thiamine pyrophosphokinase (TPK). However, TPK activity was exclusively recovered in the cytosol.

**Conclusion:**

Our results clearly indicate that mammalian peroxisomes do contain TPP but that no pyrophosphorylation of thiamine occurs in these organelles, implying that thiamine must enter the peroxisome already pyrophosphorylated. Consequently, TPP entry may depend on a specific transport system or, in a bound form, on HACL1 translocation.

## Background

Thiamine (vitamin B_1_) is a water-soluble micronutrient essential for normal cellular functions, growth and development. Humans, and other higher eukaryotes, cannot synthesize thiamine but depend on an appropriate dietary intake and absorption of this vitamin. Its plasma concentration is regulated by intestinal and renal mechanisms which play a crucial role in regulating body thiamine homeostasis. Upon entry into cells, thiamine is quickly converted to its biologically active form, thiamine pyrophosphate (TPP) by thiamine pyrophosphokinase (TPK). TPP plays a critical role in the carbohydrate and energy metabolism. It functions as a prosthetic group for the mitochondrial enzyme complexes like pyruvate dehydrogenase, α-ketoglutarate dehydrogenase and branched-chain α-keto acid dehydrogenase. In addition, TPP is involved in the cytosolic pentose pathway functioning as coenzyme for transketolase. Recently, it became clear that TPP is also important for a less well known pathway, namely the α-oxidation of 3-methyl-branched and straight chain 2-hydroxy long chain fatty acids [1-3]. In this pathway, which is confined to peroxisomes, TPP, together with Mg^2+^, is required for the proper functioning of 2-hydroxyphytanoyl-CoA lyase (2-HPCL), recently renamed to 2-hydroxyacyl-CoA lyase 1 (approved gene symbol and protein product HACL1) [4]. This peroxisomal matrix protein acts as a tetramer and catalyzes the cleavage of 2-hydroxyphytanoyl-CoA and 2-hydroxy long chain acyl-CoA into formyl-CoA and an aldehyde shortened by one carbon [1,5]. So far, HACL1 is the only known peroxisomal TPP-dependent enzyme in mammals.

The importance of TPP/thiamine in the α-oxidation pathway is stressed by the deleterious effects seen in rats given a thiamine-deficient diet enriched in phytol [3]. Phytol is the precursor of phytanic acid and is, under normal conditions, converted to pristanic acid and further β-oxidized in peroxisomes. In thiamine deficient rats, however, phytol administration results in death [3]. Presently nothing is known about the transport of thiamine or its phosphate esters across the peroxisomal membrane. Hence, we analyzed the presence of this vitamin in peroxisomes and verified whether peroxisomes are able to synthesize it starting from thiamine.

## Results and discussion

Studies on the distribution of thiamine and its phosphate esters in animal tissues appeared in the literature a few decades ago. In rat, the distribution of this vitamin has been shown to be tissue specific [6,7]. So far, most consistent data are available for rat brain [7,8], while less is known about the subcellular localization in rat liver. With regard to TPP, its content in rat brain is reported to be highest in mitochondrial and synaptosomal fractions [8], whereas in skeletal muscle [9] and rat liver [10] most TPP is reported as cytosolic.

Since the discovery of the TPP-dependent HACL1, it became clear that TPP, and in general the thiamine status of the cell [3], plays an important role in peroxisomal α-oxidation. Until now, TPP has never been measured in peroxisomes, nor has its transport over the peroxisomal membrane been considered. This study was undertaken to address these issues.

**Figure 2:**
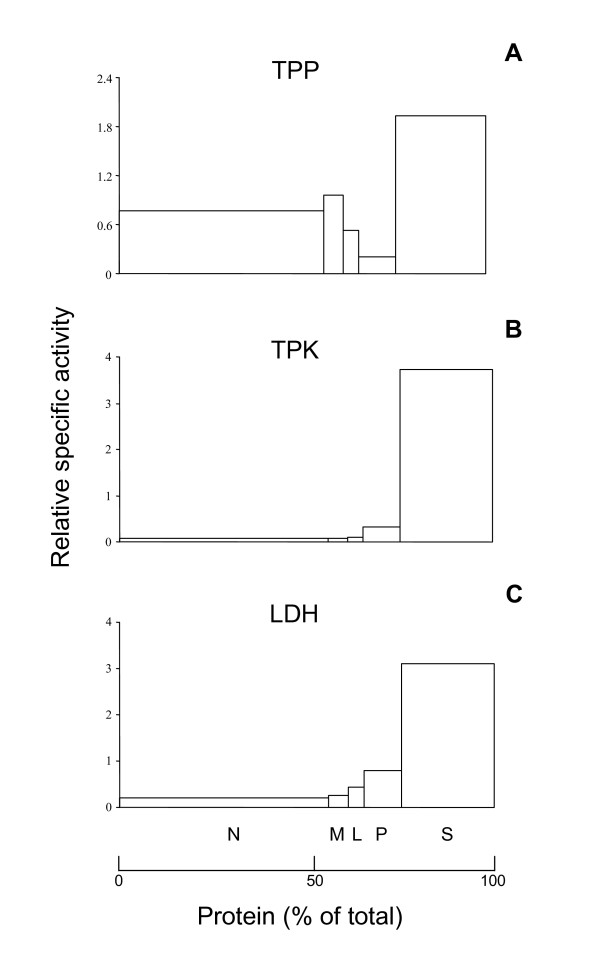
**Subcellular distribution of TPP and TPK activity**. The nuclear (N), heavy mitochondrial (M), light mitochondrial (L), microsomal (P) and cytosolic (S) fractions, obtained by fractionation of a fresh rat liver homogenate, were analyzed for TPP content (panel A), TPK activity (panel B) and lactate dehydrogenase, marker enzyme for cytosol (panel C, LDH) and other marker enzymes (data not shown). Results are expressed as relative specific activities *versus *percentage of total protein. Relative specific activity is defined as the percentage of total recovered activity present in a particular fraction divided by the corresponding percentage of protein. Recoveries for TPP content (23 nmol/g liver) and LDH (456 U/g liver) and TPK (263 nmol/g liver/h) activities were 128%, 97% and 97%, respectively. Recoveries for GDH activity and protein content were 102% and 94%, respectively (data not shown). A second fractionation (data not shown) resulted in a similar histogram for TPP (28 nmol/g liver), but with somewhat more enrichment in the M-fraction (RSA 1.79) (recovery 108%); other marker enzyme and protein recoveries varied between 70% to 102%. The TPP measured in the N-fraction is partially due to unbroken and damaged cells, which are pelleted together with nuclei.

**Figure 3:**
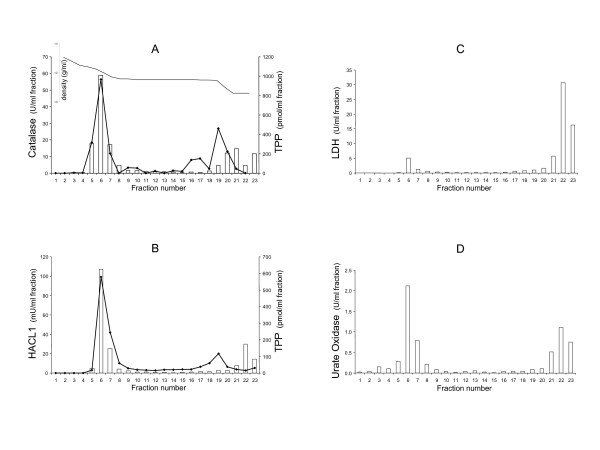
**TPP distribution in a light mitochondrial fraction after Nycodenz gradient centrifugation**. The light mitochondrial fractions obtained from rat liver were subjected to Nycodenz gradient centrifugation. Fractions were collected starting from the bottom. In a first experiment (panel A), TPP content (black line) and catalase activity (marker enzyme for the peroxisomal matrix; white bars) were measured. Recoveries for TPP content and catalase activity were 60% and 70%, respectively. Thiamine, which is also present in the L-fraction, was recovered in the top fractions (data not shown). The insert shows the density (g/ml) of the gradient. In a second experiment (panels B-D), HACL1 activity was measured (panel B; white bars; recovery 78%) revealing the highest activity (35 mU/mg protein) in fraction 6, corresponding to the TPP peak (180 pmol/mg protein, black line). In panels C and D, the distribution of LDH (marker for cytosol; recovery 121%) and urate oxidase (marker for peroxisomal core; recovery 66%) is presented (white bars).

In mouse and rat liver homogenates, prepared in the presence of phosphatase inhibitors, TPP and thiamine were found to be more abundant than TMP (data not shown). The amount of TPP measured in mouse and rat liver homogenates was 55 and 26 nmol/g liver, respectively (mean of two experiments). The latter value is comparable with previously published data [6]. When analyzing subcellular fractions of rat liver, about 50% of the TPP was recovered in the cytosol (Figure 2A), which is also in agreement with previous data [10]. The light mitochondrial fraction L, enriched in peroxisomes and lysosomes, contained only 3% of total hepatic TPP and, when related to its protein content, did not display an enrichment (Figure 2A). However, after separating the L-fraction on a Nycodenz gradient, the majority of TPP sedimented together with catalase and urate oxidase, markers for peroxisomes, to a high density (Figure 3A–D). As expected [1], HACL1 was also recovered in the peroxisomal fractions (Figure 3B). In the fraction that, based on the catalase measurement, was mostly enriched in peroxisomes, TPP increased to 177 ± 2 pmol/mg protein (mean ± SEM of three experiments), about twofold more in comparison with the L-fraction (79.4 ± 11 pmol/mg protein). Assuming that the peroxisomal compartment accounts for 2–2.5% of the total hepatic protein content [11,12] and occupies approximately 10 μl per ml of liver [13,14], one can estimate the intraperoxisomal TPP concentration at approximately 65–85 μM. As part of the TPP may leak out of peroxisomes during the fractionation procedure, this value represents a minimal estimation, but appears to be higher than the cytosolic concentration, estimated at 15–20 μM assuming that the cytosol accounts for 80% of the hepatocyte volume [13]. So on the whole, we can conclude that the peroxisome is a TPP-containing cell compartment that accounts for 2–3% of the total hepatic TPP content. Generally, isolated mammalian peroxisomes are devoid of cofactors, given the leaky nature of their membrane after tissue homogenization [15,16]. TPP would be the second example of a cofactor pool, the first one being CoA [17]. Preliminary data indicate that most of the peroxisomal TPP is not in a free form. Indeed, when freeze-thawed peroxisomes were subjected to ultrafiltration (Minicon, 10 kDa cut off membrane), only 17% of peroxisomal TPP was recovered in the ultrafiltrate. When the organelles were sonicated in the presence of 0.5% (w/v) Triton X-100 in order to disrupt the phospholipid bilayer, this value increased to 37%. These results suggest that most TPP is tightly bound to peroxisomal proteins, most likely to HACL1, or membranes. During its purification from rat liver, HACL1 appeared to gradually lose bound TPP and its activity could be restored by adding TPP and MgCl_2 _to the assay mixture [1].

The presence of TPP in peroxisomes raises the question whether thiamine or TPP is transported across the peroxisomal membrane. Transport of thiamine over the plasma membrane is performed by the high affinity carriers ThTR1 and ThTR2, encoded by the *SLC19A2 *and *SLC19A3 *gene, respectively. This transport system would also be present in the mitochondrial membrane [18] but nothing is known about a peroxisomal transporter. Uptake of thiamine would require a peroxisomal TPK to convert the vitamin into active TPP. To investigate the presence of such a kinase, we analyzed whether TPP could be formed from thiamine intraperoxisomally. The TPK activity profile overlapped with the distribution of lactate dehydrogenase (LDH) activity (Figure 2B–C). As LDH is a marker enzyme for the cytosol and TPK activity was absent or too low to be measured reliably in the Nycodenz purified peroxisomes (data not shown), we can conclude that TPK is exclusively cytosolic. This is in accordance with previous results [19,20,30] and with computer-based prediction studies, which show no peroxisome targeting signal in the primary amino acid sequence of mammalian TPK. Thus, we can conclude that peroxisomal TPP is not the product of an *in situ *pyrophosphorylation of thiamine, suggesting that TPP has to enter the peroxisome as such.

Whether TPP is transported across the peroxisomal membrane via a specific carrier has not been discovered yet. In human mitochondria, Song and Singleton [18] detected a saturable TPP transport system and the yeast mitochondrial counterpart has been functionally characterized [21]. More recently, mitochondrial TPP transport in mammals has been linked to the deoxynucleotide carrier, a protein encoded by the *SLC25A19 *gene, mutations in which cause Amish lethal microcephaly [22].

In order to better understand peroxisomal metabolism, it would certainly be useful to know more about the translocation mechanism of metabolites and cofactors across the peroxisomal membrane. So far, evidence for functional transporters in mammalian peroxisomes is limited to ATP [23] and phosphate carriers [24]. With regard to TPP, a carrier has not yet been identified, but one can also envision that the uptake of this vitamin in peroxisomes is linked with the import/tetramerization of HACL1.

## Conclusion

Using HPLC coupled with fluorimetry, we detected and measured, for the first time, the presence of TPP in purified rat liver peroxisomes. In addition, our results show that peroxisomes are devoid of thiamine pyrophosphokinase activity, which implies that vitamin B_1 _is entering the peroxisome in its diphosphorylated form. The TPP transport may be due to the existence of a specific peroxisomal TPP carrier or it may be linked, as a cofactor-protein complex, to the import of the peroxisomal TPP-dependent enzyme HACL1.

## Methods

### Materials

Thiamine hydrochloride was purchased from Janssen Chimica, TMP from Fluka and TPP from Sigma. Potassium hexacyanoferrate [K_3_Fe(CN)_6_] was purchased from Merck.

### Animals

Animal studies were approved by the University Ethics committee. Male Wistar rats, weighing approximately 200 g, and Swiss Webster mice, weighing approximately 30 g, were maintained on a constant light-dark cycle and a standard laboratory diet. Rats were fasted overnight before sacrifice.

### Preparation of homogenates and subcellular fractions

Homogenates of rat liver were prepared in 0.25 M sucrose containing 5 mM Mops-NaOH, pH 7.2, 1 mM dithiothreitol and 0.1% (v/v) ethanol (homogenization medium). Protease inhibitors were added to the homogenization medium just before use. This medium was also supplemented with phosphatase inhibitors (5 mM NaF and 50 μM orthovanadate) to prevent degradation of TPP and TMP. Subcellular fractionation into a nuclear (N), heavy mitochondrial (M), light mitochondrial (L), microsomal (P) and soluble (S) fraction was performed as described previously [25]. Fraction L was subfractionated over a Nycodenz gradient in order to obtain purified peroxisomes [26]. Marker enzymes and protein [25,27] and HACL1 [5] were measured as described previously.

### Determination of thiamine and its phosphate esters

**Figure 1:**
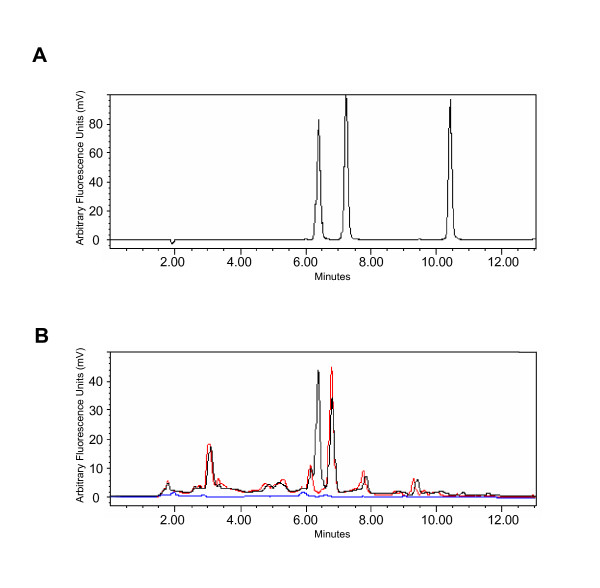
**Reversed phase separation of thiochrome derivatives of thiamine and thiamine phosphate esters**. Panel A. A mixture of derivatized standards (50 pmol each) was injected on a C18 amide column as described in Materials and Methods. The vitamins eluted in the following order: TPP (6.4 min), TMP (7.2 min) and thiamine (10.4 min). Panel B. Chromatogram of the thiochromes generated from a peroxisome enriched fraction (3.1 mg protein/ml) obtained from the Nycodenz gradient (black line), documenting the presence of TPP. Some peaks apparently originate from Nycodenz, as revealed by comparison with the profiles of the corresponding blank Nycodenz fractions, both derivatized (red line) and not derivatized (blue line).

Thiamine, TMP and TPP were analyzed by HPLC after their conversion to a strongly fluorescent thiochrome derivative by chemical oxidation in alkaline medium (adapted from [6,28]). To remove particulate matter and lipids, 100 μl sample was treated with 100 μl perchloric acid (final concentration 0.4 M) and then centrifuged at 10,000 g at 4°C for 10 min. The supernatant (100 μl) was immediately derivatized in subdued light with 20 μl 30 mM K_3 _[Fe(CN)_6_] in 15% NaOH. Methanol (10 μl) was added to increase formation of thiochromes [28]. After 60 sec, the derivatization was stopped by adding 20 μl 1 M H_3_PO_4_. Aliquots of 10 μl were injected on an Alltima HP C18 amide column (150 × 4.6 mm; 5 μm; 190Å; Alltech), connected to a Waters 1525 HPLC system coupled to a Waters 2475 multi λ fluorescence detector (excitation and emission wavelength 367 and 435 nm, respectively). The mobile phase (1 ml/min) consisted of a gradient of K-phosphate buffer (140 mM, pH 7)/12% methanol (buffer A) to 70% methanol (buffer B). The analytical run started with 100% buffer A; within 10 min, the ratio A/B reached 50/50, becoming 0/100 in the following 5 min. Using this gradient, TPP, TMP and thiamine could be well separated (Figure 1A). The addition of 1.5% N,N-dimethylformamide (DMF) to buffer A, reported to result in better separation [29], improved neither the separation nor the peak shape of the three thiochromes in our hands. Moreover, the baseline increased during elution when DMF was present.

To investigate the stability of TPP in homogenates, mouse liver was homogenized in sucrose medium and stored at -20°C for 5 days. When prepared in the absence of phosphatase inhibitors, TPP levels dropped by approximately 42%, compared to the use of medium with inhibitors. The amount of TPP measured in perchloric liver extracts or homogenates made in the presence of inhibitors was comparable.

When analyzing samples containing Nycodenz, the baseline was less stable and aberrant peaks were noticed that interfered with the accurate determination of low levels of TPP (< 1 pmol/sample). Apparently, these peaks originated from the tri-iodinated benzoate compound, but their exact nature was unclear (Figure 1B). When required, data were corrected by analyzing the corresponding fractions collected from blank gradients.

### Thiamine pyrophosphokinase activity measurement

Thiamine pyrophosphokinase activity was measured in rat liver fractions. Samples (50 μl) were incubated at 37°C with 8 mM thiamine, 24 mM ATP, 8 mM MgSO_4 _and 40 mM Na-phosphate buffer, pH 7.4 containing 5 mM NaF and 0.1 mM orthovanadate as phosphatase inhibitors (adapted from [30]). Final volume was 200 μl. After 1 h incubation, 100 μl sample was treated with 100 μl perchloric acid and further processed as described above to establish the amount of TPP formed. Values were corrected for endogenous TPP by omitting thiamine from the reaction mixture and used to calculate TPK activity, which was expressed as nmol TPP produced/mg protein/h at 37°C.

## Abbreviations

HACL1: 2-hydroxyacyl-CoA lyase 1

TPP: thiamine pyrophosphate

TPK: thiamine pyrophosphokinase

TMP: thiamine monophosphate

DMF: N,N-dimethylformamide

## Authors' contributions

PF carried out the rat liver fractionations, biochemical assays, chromatographic measurements and drafted the manuscript. MS participated in the optimization of the chromatographic measurements and critically revised the manuscript. MC and PPVV conceived the study and coordinated the experiments and preparation of the manuscript. All authors read and approved the final manuscript.
